# Understanding the Pathway of Cancer Information Seeking: Cancer Information Services as a Supplement to Information from Other Sources

**DOI:** 10.1007/s13187-021-02095-y

**Published:** 2021-11-16

**Authors:** Doreen Reifegerste, Magdalena Rosset, Fabian Czerwinski, Eva Baumann, Andrea Gaisser, Evelyn Kludt, Susanne Weg-Remers

**Affiliations:** 1 School of Public Health, Bielefeld University, Universitätsstr. 25, 33615 Bielefeld, Germany; 2 Department of Journalism and Communication Research, Hanover University of Music, Drama and Media, Expo Plaza 12, 30539 Hannover, Germany; 3 Cancer Information Service, German Cancer Research Center, Im Neuenheimer Feld 280, 69120 Heidelberg, Germany

**Keywords:** Cancer, Cancer information seeking, Cancer information service, Information sources, Pathway

## Abstract

Cancer information services (CISs) can play an important role within the pathway of cancer information seeking, but so far, this role is not well understood. Callers (*n* = 6,255) who contacted the largest provider of cancer information in Germany participated in a survey in which they reported their information sources, information level, and needs leading to the call. Persons with prior information from a physician (*n* = 1,507) were compared to people with prior online information (*n* = 901) and people with prior information from both sources (*n* = 2,776). Nearly all callers (96.7%) stated prior sources, while physicians and the Internet were the most frequently reported sources. People, who only talked to a doctor before, are more likely to be a patient and in the disease stages during/after the first treatment or with recurrence than prior Internet users. The two groups do not differ in their prior information level but did differ in their information needs. CISs are an important supplement to other sources, while the information repertoire depends on patients’ stages during the cancer journey. Specific characteristics and needs of callers with different prior information sources help to individualize the service of CISs and similar providers.

## Introduction

After a cancer diagnosis, patients and their family members often need information and advice to help them cope with the situation and related challenges. They are confronted with many uncertainties and have to make treatment decisions or support patients [1, 2].

One strategy of managing and coping with these uncertainties is to actively seek cancer-related health information from various sources, such as doctors, the Internet, other media, support groups, and (to a lesser extent) cancer information services (CISs) [3]. In a scoping review of cancer information studies from 2007 to 2017 comparing the preferences for various cancer information channels, it was found that doctors ranked first among patients and caregivers, and the Internet second [4, 5]. As physicians are no longer the only source of health information, it also seems necessary to look at the sequence of sources people turn to in more detail. Pathways of information seeking within the repertoire of information sources are often looked at within the context of medical consultations [6]. So we already know that online sources are often used to prepare, or follow-up face-to-face encounters with physicians to complement unfulfilled needs and gain autonomy [7]. However, we know little about the role of further information sources, such as CISs, within these pathways.

Understanding the specific characteristics and needs of callers with prior information from different sources can be helpful to better fulfill their individual information and support needs and improve the services of CISs and other cancer-related information providers. The results of such a study could also serve as a basis for the development of communication strategies and tailored information resources for helpline operators and medical professionals [8].

Physicians are an important source of information about a diagnosis, treatment, and prognosis [5]. Cancer information from health professionals is trusted most [9], and most people prefer cancer-related information from health professionals [4]. However, doctors often work under rigid time constraints, keeping them from an in-depth conversation with their patients. Some might lack communication skills that encourage patients to talk about psychological concerns [10] or other support needs. Thus, cancer patients are often dissatisfied with how doctors and other health care professionals respond to their information needs [11], see their information demands unmet [1], or have problems expressing their feelings and concerns to their doctors [10]. In addition, family members often feel that their information needs are not taken seriously by medical staff and often face difficulties retrieving information from health professionals [12]. Thus, their information needs (in addition to those of the patients) often remain unfulfilled [2, 13–15].

In contrast, the Internet has increased health and medical information availability in recent decades, providing patients and caregivers with plenty of opportunities to search for cancer-related information [5]. When looking for health information, most people turn to the Internet first [16]. But the multitude of online information and the multiple sources can make it harder for patients and caregivers to find reliable information and properly evaluate (possibly conflicting) information.

Preferences for and evaluation of cancer information from the Internet seem to differ depending on the individual’s role as a patient or a caregiver. Although caregivers prefer doctors as an information source, they still mostly use the Internet to obtain information for specific problems [17] and more likely to use the Internet for cancer information than patients [18]. Moreover, they are more satisfied with the Internet as a source for cancer-related information than patients, while patients show higher satisfaction with doctors [19]. An explanation for these results could be that while patients directly contact doctors and other health professionals, their family members and friends often receive much less attention and support [20]. Family members face particular challenges gaining health information and counseling. Still, they are not the primary target group of health care providers, so they depend on additional sources to fulfill their informational needs and actively seek cancer-related information complementary to the information they gain from health professionals [17, 21].

Taken together, different cancer information seekers face different informational challenges, and either source leaves unmet needs for both patients and caregivers. Thus, additional sources that are available and reliable to supplement the primary sources of cancer information are needed. Accordingly, CISs, in addition to medical staff and other information sources, have become established as easily accessible sources for individually tailored, valuable, and reliable (i.e., evidence-based) information and counseling [22]. CISs are available in many parts of the world. They offer informational and emotional support for patients and their family members and friends via telephone, e-mail, social media, or letter [23]. From CIS evaluation surveys around the world, it is known that people contacting CISs are overall satisfied with the information they receive [23] and that use of the service mostly provides several cognitive and emotional benefits for them [22, 24, 25].

Studies show that cancer survivors use about five different information sources on average when searching for cancer-related information [5, 26], and 69% of cancer patients obtain information from other sources besides their health care providers [27]. This suggests that information from the Internet or the physician alone does not comprehensively satisfy patients’ emotional and informational support needs. Such unmet needs can be a predictor for using alternative information sources to gratify remaining information needs [28]. Also, it seems necessary to understand usage patterns of such sources over time [26]. Hence, CISs may supplement information retrieved from other sources used before the CIS call. This assumption is supported by the fact that some CIS callers (26.3%) had already sought cancer-related information from the Internet before contacting the CIS [29].

So far, prior information behavior has scarcely been considered when analyzing CIS callers because most existing research focuses on using different single sources and does not consider the supplementary role of CISs in the seeking pathway within the repertoire of information sources of cancer patients and their caregivers. Accordingly, to better understand the cancer information journeys of patients and their caregivers and improve the adjustment of information provision and counseling by health care providers and CISs, the personal and needs-related differences of callers with prior information from different sources must be analyzed.

This requires to take into consideration the already known determinants that help to explain the use and preference for health professionals and the Internet as the two major information sources, i.e., the accessibility of different information sources for different groups of information seekers, socio-demographic and cancer-related characteristics, and information-related factors. Studies show that older people are more likely to seek information on cancer than younger people overall [30, 31]. Focusing on the Internet reveals that more younger people prefer to seek cancer-related information on the Internet than older people. In comparison, more older people prefer cancer-related information from health professionals than younger people [30–34]. Higher educated people would rather seek cancer-related information on the Internet [30, 33], while persons with a lower level of education turn more to health professionals [32]. In addition to age and education, sex is also associated with cancer information seeking. Women are more likely to seek cancer-related information than men [30, 31, 35–37] and would also contact CISs more often than men [38].

Furthermore, certain cancer-related characteristics can be associated with the information-seeking behaviors of patients and their family members. For example, the information needs of patients and family members are often associated with the cancer stage of the patient and differ throughout the disease [14, 39–44].

When looking at the information needs of people contacting a CIS, the most relevant topics are treatment, prognosis, general cancer information, and psychosocial support; among caregivers, the need for psychosocial support and prevention rank higher than among patients [4].

Hence, users of a CIS appear to have different information needs. They might also differ regarding their perceived prior information level after using different sources — particularly from a doctor or from the Internet — before contacting a CIS. They might be already informed to a certain degree, and it seems very likely that differences in the requests to CISs arise from the use of different sources. But there is little research on individual characteristics explaining the differences between those who gather information from other sources before turning to a CIS. Also, it can be expected that patients and family members who contact CISs differ regarding their support needs depending on the information they have obtained from other sources before. Therefore, this study analyzes CIS callers’ prior information sources before contacting the CIS and determinants associated with prior information from the different sources. Thus, our first research question addresses the type of these prior information sources.

RQ1: Which prior information sources do CIS callers have?

As health professionals and the Internet are the two major prior information sources before people call a CIS, the specific research questions regarding the comparison are as follows:

RQ2: How do CIS callers with different prior information sources differ regarding (a) socio-demographics (sex, age, education), (b) cancer-related characteristics (cancer stage, type of caller — patient or family member), and (c) information-related factors (perceived level of prior information, informational needs, use of further information sources after their contact with the CIS)?

In addition, we aimed to analyze the relative impact of these three types of factors:

RQ3: What is the additional explanation value of the three-factor groups?

## Method

### Procedure and Participants

The analysis is based on two large-scale surveys of users of the telephone service of the largest provider of cancer information in Germany, the Krebsinformationsdienst (KID), conducted between April and September 2011 and between June 2016 and April 2017. The KID is a division of the German Cancer Research Center. The service is publicly funded, free of charge, comprehensive, and understandable. The KID also provides information for the general public (e.g., individuals seeking cancer prevention or early detection), journalists, or health care professionals [45]. Callers who identified themselves as any of these were excluded from the analysis.

Thus, only callers being patients or family members of patients were included in the analysis. Those who agreed to participate (*n* = 4,715 callers between April and September 2011; *n* = 4,713 callers between June 2016 and April 2017) received a questionnaire (2011: postal, 2016: postal or online) within 2 weeks after the contact with KID. The response rate was 67.6% (*n* = 3,185) in 2011, and 65.2% (*n* = 3,070) in 2016, where 65.2% participated in the postal and 34.8% in the online survey. Thus, in total, *n* = 6,255 callers participated across the two surveys.

### Measures

From the callers’ dataset, sex (0 = female; 1 = male), age (open), education (0 = below university entrance degree; 1 = university entrance degree), type of caller (0 = caregiver; 1 = patient), and stages of cancer were included. The different stages of the disease are described as five phases from cancer diagnosis to palliative care (diagnosis, during the first treatment, after the first treatment, recurrence, and palliative stage). Anonymous data on all KID users and, if applicable, the respective patient, including data on socio-demographic and cancer-related characteristics, were available from the routine CIS contact documentation in an electronic database that serves statistical and quality management purposes. An exception from ethical clearance is granted by the data protection authority for this type of data collection, as callers use KID anonymously, and only nonidentifiable data are collected.

The questionnaire was in German. Perceived level of information before the call was measured with the question: “How informed did you feel before calling the KID?” (1 = well; 2 = not very well; and 3 = not informed at all). We recorded the answers to 0 = not (well) informed and 1 = well informed because only very few callers (4%) reported being completely uninformed.

Information needs and concerns were assessed by giving a list of possible answers to the question: “What did you need when you contacted the CIS?”. Respondents could endorse each of the following: general explanation, case-specific information, case-specific decisional support, emotional support, and contact information for further support. To analyze the callers’ prior information source before contacting the CIS, they were asked to state the previously used sources of information (talks with health professionals, talks with family/friends, other patients, counseling services, TV/radio, print media, Internet, online support groups).

Use of further information sources after the call was measured with the statement: “I have/my family member has used or intent/s to use the provided contact for further information.” Because the questionnaire was sent out at least some days after the call, the statement included both the actual behavior and the intention but always refers to using the provided contact after the call.

### Data Analysis

To address RQ2, we performed simple statistical independence tests between the three groups across all variables that were later used in the regression model. The vast majority of variables were proportions, so we used Cramer’s *V* to test significant differences among the groups. To test the influence of age, we used a simple ANOVA *F*-test to test the mean differences.

For the logistic regression, we only included callers, who either stated using both doctors and the Internet (*n* = 2,776) as well as those persons with prior information only from doctors (*n* = 1,507) and people with prior online information only (*n* = 901).

The category “Internet-only” was applied to all callers who reported prior information only from online resources. The category “doctor only” was equally applied to all callers who reported their prior information stems from their doctor only (general practitioner and/or specialist/oncologist). Last, the category “both Internet and doctor” was applied to all respondents who reported to have been prior informed by online information as well as from their doctors. We excluded respondents who also used other (*n* = 862) or no sources of prior information (*n* = 209) to avoid confounding influences and to focus on the groups with the two most frequently used prior information sources.

This resulted in a final sample of *n* = 5,184 for the hierarchical multivariable logistic regression analysis, which was conducted to predict the type of prior information (doctor vs. Internet vs. both).

As independent variables, we included sex, age, and education as socio-demographic variables in the first block. After including the cancer-related characteristics (patient’s stage of cancer and type of caller) in the second block, the third block (information-related factors) included prior information level, information demands, and the use of further information sources. Data for 2011 and 2016 were analyzed together as the results were very similar. This was included in the model as a control variable to control for possible differences due to the year of data collection. All analyses have been conducted using SPSS® 26.

## Results

Concerning RQ1, 96.7% used different information sources before they turned to the CIS. The average number of prior sources was *M* = 3.73 (*SD* = 1.94), while the physicians and the Internet were the most frequently used sources (Table 1).

**Table 1:** Prior information sources before contacting the cancer information service (*N* = 6.255)

	%	n
Medical specialist	71.8	4,491
Oncologist	65.6	4,103
Internet	60.6	3,791
General practitioner	43.1	2,696
Books/brochures	35.7	2,233
Family members/friends	33.6	2,102
Newspapers/magazines	19.9	1,245
Television/radio	17.5	1,095
Social support groups	12.2	763
Customer magazines	9.8	613
Cancer information service	9.6	600
Health insurance	5.8	363
Others	8.9	557

The age of respondents who used the Internet and/or physicians as prior sources ranged from 20 to 96 years (*M* = 58.1; *SD* = 13.1); the majority were female (65.5%) and had a higher education level (52.5%). About 35% of respondents were family members or friends who contacted the CIS on behalf of patients. The most relevant information needs were case-specific explanations (85.5%), case-specific decisional support (72.0%), and additional sources of support (20.2%) (Table 2).

**Table 2:** Descriptive statistics

Category	Variable	Group 1: information from both the Internet and from a physician (*n* = 2,776)	Group 2: information from the Internet, but not from a physician (*n* = 901)	Group 3: Information from a physician, but not from the Internet (*n* = 1,507)	Total (*n* = 5,184)	Group differences
Socio-demographics	Sex of caller: male (%)	35.6	32.4	33.7	34.5	*V* = .03
	Age of caller in years (mean [SD])	56.8 (12.6)	53.3 (14.0)	63.6 (12.2)	58.1 (13.3)	*F* = 213.3***
	High level of education (%)	57.3	58.4	40.0	52.5	*V* = .16***
Cancer stage	Diagnosis (%)	17.7	25.2	14.2	18.0	*V* = .09***
	First treatment (%)	31.1	26.1	26.2	28.8	
	After first treatment (%)	24.0	18.2	29.1	24.5	
	Recurrence (%)	22.5	23.5	25.5	23.6	
	Palliative (%)	4.6	7.0	5.0	5.2	
Type of caller	Patient (%)	68.4	33.9	77.4	65.0	*V* = .31***
Prior-info level	Perceived level of pre-information = good (%)	54.8	46.0	49.7	51.8	*V* = .07***
Informational needs	General explanations (%)	17.4	16.9	19.6	18.0	*V* = .03
	Case-specific explanations (%)	88.1	84.2	81.6	85.5	*V* = .08***
	Case-specific decision support (%)	76.5	61.5	70.0	72.0	*V* = .13***
	Additional sources of support (%)	19.9	27.1	16.7	20.2	*V* = .09***
	Emotional support (%)	12.0	13.3	13.1	12.5	*V* = .02
Support/further actions	Used further informational sources after the call (%)	46.7	46.7	36.0	43.8	*V* = .10***
Data collection	(% Online–2016 only)	73.7	79.6	49.3	67.7	*V* = .25***
	Year of data collection (2011 in %)	50.5	52.6	52.1	51.3	*V* = .02

In case of proportions, we used Cramer’s *V*; in case of means (age only), we used ANOVA (*F*). Type of caller includes patient vs. family members

^*^p < .05 **p < .01 ***p < .001

Concerning RQ2, the comparison of descriptive results between the three groups of callers (doctor vs. Internet vs. both as the prior information source) revealed significant differences for some characteristics. Internet-informed callers are significantly younger (53.3 vs. 63.6 years) and better educated (58.4% vs. 40.0% with university entrance degree) than physician-informed callers (both *p* < 0.001). Furthermore, for Internet-informed callers, the patient’s cancer stage is much more likely to be in the very first (diagnosis) or last (palliative) stage. The Internet-informed callers are significantly more often surrogate seekers (i.e., family members or friends) than the physician-informed callers (66.1% vs. 22.6%; *p* < 0.001) and tend to need more additional sources of support (like brochures, written information material or further contact addresses; 27.1% vs. 16.7%) but less case-specific support (61.5% vs. 70.0%; both *p* < 0.001). Finally, they stated more frequently to have used further information sources after their first contact with the CIS (46.7% vs. 36.0%; *p* < 0.001).

The results of the logistic regression models (Nagelkerke’s *R*^2^ = 20.8%) indicate that the demographic variables (RQ2a) are only significant in the full model when comparing users of both sources with only doctor users. Being female (*OR* = 1.477, *p* < 0.001), being older (*OR* per year: 1.043, *p* < 0.001), and lower educated (*OR* = 1.597, *p* < 0.001) increases the likelihood that the prior information source was only a doctor (Table 3). In contrast, there are no significant associations when comparing only Internet users and users of both prior sources.

**Table 3:** Results of the logistic regression model (DV: source of prior info level^a^)

	Category	Variable	Block 1		Block 2		Block 3	
			OR	95% CI	OR	95% CI	OR	95% CI
Only Internet vs. both doctor and Internet	Socio-demographics	Sex of caller (female)	1.116	[.91; 1.37]	1.240*	[1.00; 1.53]	1.228	[.99; 1.52]
		Age of caller (in years)	.980***	[.97; .99]	.995	[.99; 1.00]	.996	[.99; 1.00]
		Level of education (low/middle)	.897	[.74; 1.09]	.941	[.77; 1.15]	.939	[.77; 1.15]
	Cancer stage	Ref.: Diagnosis						
		First treatment			.677**	[.52; .89]	.710*	[.54; .94]
		After first treatment			.909	[.67; 1.23]	.905	[.67; 1.23]
		Recurrence			.868	[.65; 1.16]	.901	[.68; 1.20]
		Palliative			.846	[.55; 1.31]	.856	[.55; 1.33]
	Type of caller	Surrogate seeker			4.318***	[3.50; 5.33]	4.071***	[3.29; 5.04]
	Prior-info level	Perceived level of pre-information (no/less good)					1.206	[.99; 1.47]
	Informational needs	General explanations					.895	[.69; 1.16]
		Case-specific explanations					.795	[.59; 1.07]
		Case-specific decision support					.561***	[.45; .69]
		Additional sources of support					1.138	[.91; 1.43]
		Emotional support					1.113	[.83; 1.50]
	Further actions	Used further informational sources					.970	[.80; 1.18]
Only doctor vs. both doctor and Internet	Socio-demographics	Sex of caller (female)	1.518***	[1.27; 1.81]	1.507***	[1.26; 1.81]	1.477***	[1.23; 1.78]
		Age of caller (in years)	1.046***	[1.04; 1.05]	1.044***	[1.04; 1.05]	1.043***	[1.04; 1.05]
		Level of education (low/middle)	1.707***	[1.45; 2.00]	1.678***	[1.43; 1.97]	1.597***	[1.36; 1.88]
	Cancer stage	Ref.: Diagnosis						
		First treatment			1.185	[.91; 1.54]	1.184	[.91; 1.55]
		After first treatment			1.429**	[1.10; 1.87]	1.397*	[1.07; 1.83]
		Recurrence			1.349*	[1.03; 1.76]	1.385*	[1.06; 1.82]
		Palliative			1.227	[.79; 1.91]	1.270	[.81; 1.99]
	Type of caller	Surrogate seeker			.764**	[.63; .93]	.746*	[.61; .91]
	Prior-info level	Perceived level of pre-information (no/less good)					1.313**	[1.11; 1.55]
	Informational needs	General explanations					1.184	[.96; 1.47]
		Case-specific explanations					.680**	[.54; .86]
		Case-specific decision support					.698***	[.58; .84]
		Additional sources of support					.808	[.65; 1.01]
		Emotional support					.963	[.75; 1.24]
	Further actions	Used further informational sources					.705***	[.60; .83]
*Nagelkerke’s R*^2^ *(ΔR*^*2*^*)*			10.3		18.5 (8.2)		20.8 (2.3)	

Missing cases were deleted listwise, resulting in *n* = 3,558. *OR*, odds ratio; *CI*, confidence interval. All models controlled for the impact of the year of data collection (all n.s.)

^a^Coding of the dependent variable: 0 = both doctor and Internet (reference category); 1 = only Internet; 2 = only doctor

^*^p < .05 **p < .01 ***p < .001

The results for cancer-related characteristics provide answers to RQ2b. Surrogate seekers (in contrast to patients) are more likely to belong to the group of Internet-informed callers who either only used the Internet before the CIS contact (*OR* = 4.071, *p* < 0.001) or who had both the Internet and the doctor as prior information sources (*OR* = 0.746, *p* < 0.05). Some stages of the cancer trajectory (during or after the first treatment and recurrence) are also strongly associated with prior information sources of the caller.

Concerning RQ2c, some of the information-related factors were associated with the prior information source. While the two groups — users of the Internet and users of both sources — do not differ in their perceived level of prior information, the respondents who felt less well informed were more likely to be informed only by physicians (*OR* = 1.313, *p* < 0.01).

Further significant differences could be identified for callers’ informational demands. Seeking case-specific explanations (*OR* = 0.680, *p* < 0.01) and case-specific decisional support (*OR* = 0.698, *p* < 0.001) is much more likely among users of both prior sources compared to physician-informed callers and compared to only online information seekers (*OR* = 0.561, *p* < 0.001).

People with prior online and physician information are significantly more likely to report to have used further information sources after the call to the CIS than physician-only informed callers (*OR* = 0.705, *p* < 0.001).

Overall, the introduction of cancer-related characteristics in model 2 led to a considerable increase in the pseudo-*R*^2^ value (from 10.3 to 18.5%). In contrast, the addition of informational demands and further actions following the contact to the CIS resulted only in a minor advancement regarding this characteristic (from 18.5 to 20.8%).

## Discussion and Conclusion

### Discussion

The current study aimed to understand the complementary role of CIS by investigating the sources that patients or relatives have sought before the contact with the CIS and to analyze the factors influencing the pathways of information sources. Nearly all respondents used other sources before contacting a CIS. This fact and the high number of prior sources demonstrate that the CIS serves as an important supplement to information from other sources. Physicians and the Internet were the most frequently used prior sources, which was not surprising as both are popular sources for cancer information [4]. However, the differences between callers with prior information from a doctor and/or from the Internet regarding socio-demographic variables, cancer-related factors, and information-related factors might explain specific informational needs and gratifications sought from CISs.

Regarding the influence of socio-demographic variables, our results are in line with existing research. Older individuals are more likely to seek information from health professionals [30–34], women more often seek cancer-related information than men independent of the source [30, 31, 35–37], and higher educated persons are more likely to have prior cancer information from the Internet [30, 33].

Regarding cancer-related characteristics, the results regarding the type of caller confirm that patients rather have access to doctors. Thus, they are more likely to have prior information from a physician, while family members are more likely to have used the Internet before contacting a CIS [4]. Our results extend existing research by showing that different stages of the disease seem to be associated with different prior information sources.

Regarding the informational needs of callers to the CIS, it has to be noted that the most relevant informational needs in all groups under investigation are case-specific explanations and decision support. Although there are differences for users on different pathways within the repertoire of cancer information, this indicates that the main function of the CISs’ supplementary role is to provide case-specific support. However, in the case of prior information from doctors, this seems surprising as doctors are supposed to provide such case-specific explanations.

The relative explanation value of the three-factor groups suggests that socio-demographics and cancer-related characteristics are the main determinants of information demands and behavior on the cancer information-seeking pathway.

### Conclusion

In summary, our study provides an extension to existing research because of its focus on the process of cancer-related information gathering, while other studies often only focus on contact with the CIS. The fact that nearly all surveyed CIS callers had sought or received information before their counseling requests emphasizes the complementary role of the CIS. This study shows that it is important to consider the sequence and combination of sources within the repertoire of information sources instead of assuming and analyzing their exclusive selection in theoretical concepts and empirical design.

The strengths of the present study are its large sample size and the inclusion of data not only from patients but also from surrogate information seekers. However, like any secondary analysis, the data also has some limitations, which should be considered when interpreting the results. First, this study focused on prior information from a doctor and/or from the Internet of CIS callers. Additional research on using other information sources or combining different information sources (also those without contact with the CIS) is required to see how different sources complement each other. Second, this study does not consider whether callers used further information sources after contacting the CIS. We have not analyzed which specific information sources they used afterward and which information demands they had in doing so. Future research should analyze the complete information-seeking pathway within the information repertoire, including interpersonal informal and professional sources and different mobile and social media formats. Third, the lower percentage of participants in the online version of the survey indicates that people who took part in the survey preferred the postal version (2016). Thus, future studies should also check how callers could be motivated to fill in the online version more often.

### Practice Implications

Understanding the specific characteristics and demands of callers with prior information from different sources can help better meet their individual information and support needs and improve the service of CISs and other cancer-related information providers. This could serve as a basis for the development of communication strategies and tailored information resources for CISs. Although most CIS callers are already quite satisfied with the service, perceive information as helpful, and recommend the service to others, a more tailored pathway could improve the cancer information status of patients and their family members.

Insights into differences between callers with different sources of prior information can facilitate the provision of cancer-related information to CIS users and deduce suggestions for doctors to proactively recommend further information sources (e.g., the contact information of a CIS). A study among German practitioners (not oncologists) in 2011 indicated that only about two-thirds know about the CIS, only about 20% called themselves, and only about 30% recommend it to their patients [46]. So far, most CIS users learn about the service from the Internet, and only about 10% from their health professionals [47]. This might be especially relevant when patients still have unmet information needs, require further explanations, decisional support, or emotionally overwhelmed in the consultation with a physician and thus unable to assimilate all the information discussed. These data show the potential of CISs to support patients and caregivers in understanding cancer-related information and make informed decisions. Similarly, Internet information could also enlist the CIS and other contact addresses as further sources of support and, thus, actively support the pathway of cancer information seeking.
